# Supplementary material for: Cryptococcus neoformans Prp8 Intein: An In Vivo Target-Based Drug Screening System in Saccharomyces cerevisiae to Identify Protein Splicing Inhibitors and Explore Its Dynamics
Source: J Fungi (Basel). 2022 Aug 12;8(8):846. doi: 10.3390/jof8080846 (PMC9410109; doi:10.3390/jof8080846)
Supplement: Supplementary file 1 [file jof-08-00846-s001.zip › jof-1778599-supplementary.pdf]

Supplementary Materials

Cryptococcus neoformans Prp8 Intein: An In Vivo Target-Based Drug Screening System in *Saccharomyces cerevisiae* to Identify Protein Splicing Inhibitors and Explore Its Dynamics

José Alex Lourenço Fernandes [1,2,3,*], Matheus da Silva Zatti [1,2], Thales Domingos Arantes [4], Maria Fernanda Bezerra de Souza [2], Mariana Marchi Santoni [5], Danuza Rossi [6], Cleslei Fernando Zanelli [5], Xiang-Qin Liu [7], Eduardo Bagagli [8] and Raquel Cordeiro Theodoro [1,*]

[1] Institute of Tropical Medicine, Federal University of Rio Grande do Norte (UFRN), Natal 59077-080, Rio Grande do Norte, Brazil
[2] Department of Biochemistry, Biosciences Center, Federal University of Rio Grande do Norte (UFRN), Natal 59078-900, Rio Grande do Norte, Brazil
[3] Ottawa Hospital Research Institute (OHRI), The University of Ottawa, Ottawa, ON K1H 8M5, Canada
[4] Department of Biosciences and Technology, Institute of Tropical Pathology and Public Health, Federal University of Goiás, Goiânia 74605-050, Goiás, Brazil
[5] School of Pharmaceutical Sciences, São Paulo State University (UNESP), Araraquara 14800-903, São Paulo, Brazil
[6] Pensabio, São Paulo 05005-010, São Paulo, Brazil
[7] Department of Biochemistry and Molecular Biology, Dalhousie University, Halifax, NS B3H 4R2, Canada
[8] Microbiology and Immunology Department, Biosciences Institute of Botucatu, São Paulo State University (UNESP), Botucatu 18618-689, São Paulo, Brazil
* Correspondence: alfernandes@ohri.ca (J.A.L.F.); raquel.theodoro@ufrn.br (R.C.T.)

Supplementary Materials

Figure S1 – 3D structure of the *S. cerevisiae* Ura3 dimer and CnePrp8i insertion site. 2

Table S1 – Primers used in this work 3

Figure S2 – pRS313 Plasmid Map and Sequence 4

Figure S3 – pRHis Plasmid Map and Sequence 7

Figure S4 – pRExt Plasmid Map and Sequence 10

Figure S5 – pRInt Plasmid Map and Sequence 13

Figure S6 – pRMut Plasmid Map and Sequence 16

Figure S7 – Alignment of the Proteins generated by this work, highlighting its modifications 19

Figure S8 – Protein sequences generated by this work, highlighting its modifications 20

Figure S1 – 3D structure of the *S. cerevisiae* Ura3 dimer and CnePrp8i insertion site.

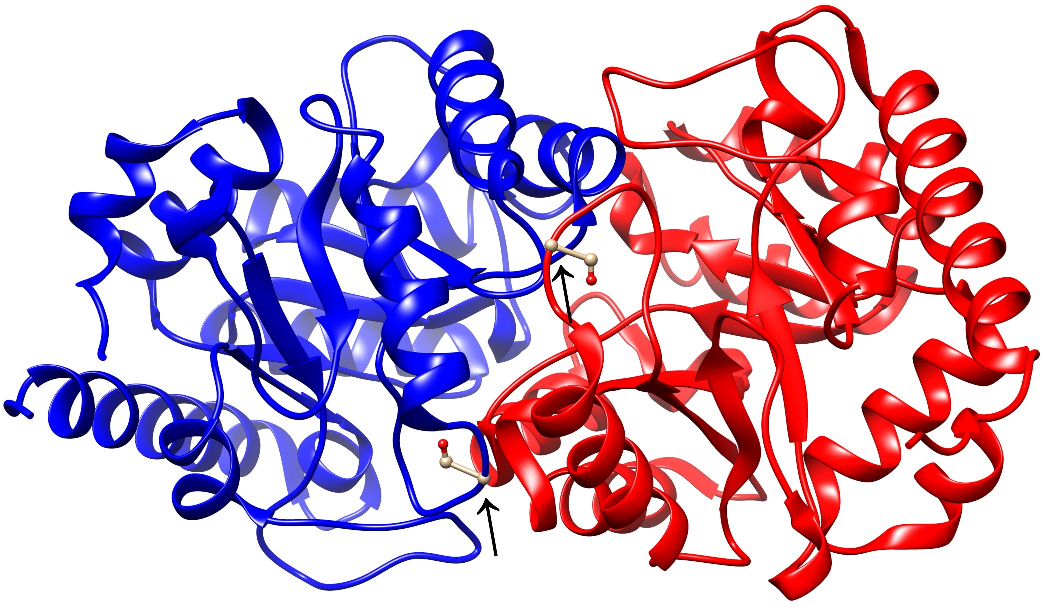

CnePrp8i insertion site (black arrows) and Serine 158 have been highlighted. Based on X-ray diffraction crystal structure from PDB 1DQX – Orotidine 5'-phosphate Decarboxylase complexed to 6-hydroxyuridine 5'-phosphate (BMP) at 2.40 Å resolution. For the purpose of this figure, BMP molecules have been removed.

Table S1 – Primers used in this work

#	Primer Name	Sequence 5′ → 3′	Modification	T_a (°C) *	Use
Set 1	M13F	GTAAAACGACGGCCAGT	-	55	Insert cloning amplification using the plasmid backbone
	M13R	AACAGCTATGACCATG	-		
Set 2	pCnePrp8-Intein_F	TGTCTGCAGAATGGTACTCG	-	55	CnePRP8i amplification, used for RT-PCR
	pCnePrp8-Intein_R	CAATACCAAATAGTCATGACG	-		
Set 3	P35	CGCGGATCCGCTTTTCAATTCAATTCATC	-	59	Insert amplification using the URA3 promoter and URA3 terminal site
	P102	CGCAAGCTTTTAGTTTTGCTGGCCGCATCTTC	-		
Set 4	p3s	ATGTCGAAAGCTACATATAAGG	-	62	Insert amplification using the URA3 initial and terminal sites, used for RT-PCR.
	p102s	TTAGTTTTGCTGGCCGCATCTTC	-		
Set 5	ppRCT2(C→A)_1F	GCTCTGCAGAATGGTACTCG	5' phosphorylated	65	pRInt whole plasmid amplification, used for site-directed mutagenesis
	ppRCT2(C→A)_1R	GGCCTTGCATGACAATTCTG	5' phosphorylated		
Set 6	ppRCT2(N→A)_2F	TCCGGCCTATCTACTGGAGA	5' phosphorylated	65	pRInt whole plasmid amplification, used for site-directed mutagenesis
	ppRCT2(N→A)_2R	AGCGTGCAATACCAAATAGTCA	5' phosphorylated		
Set 7	Mut1F	AGAATTGTCATGCAAGGCCGC	-	57	Identification of site-directed mutagenesis, used the screen ligated plasmids
	Mut2R	CCAGTAGATAGGCCGGAAGC	-		

* Annealing Temperature.

Figure S2 – pRS313 Plasmid Map and Sequence

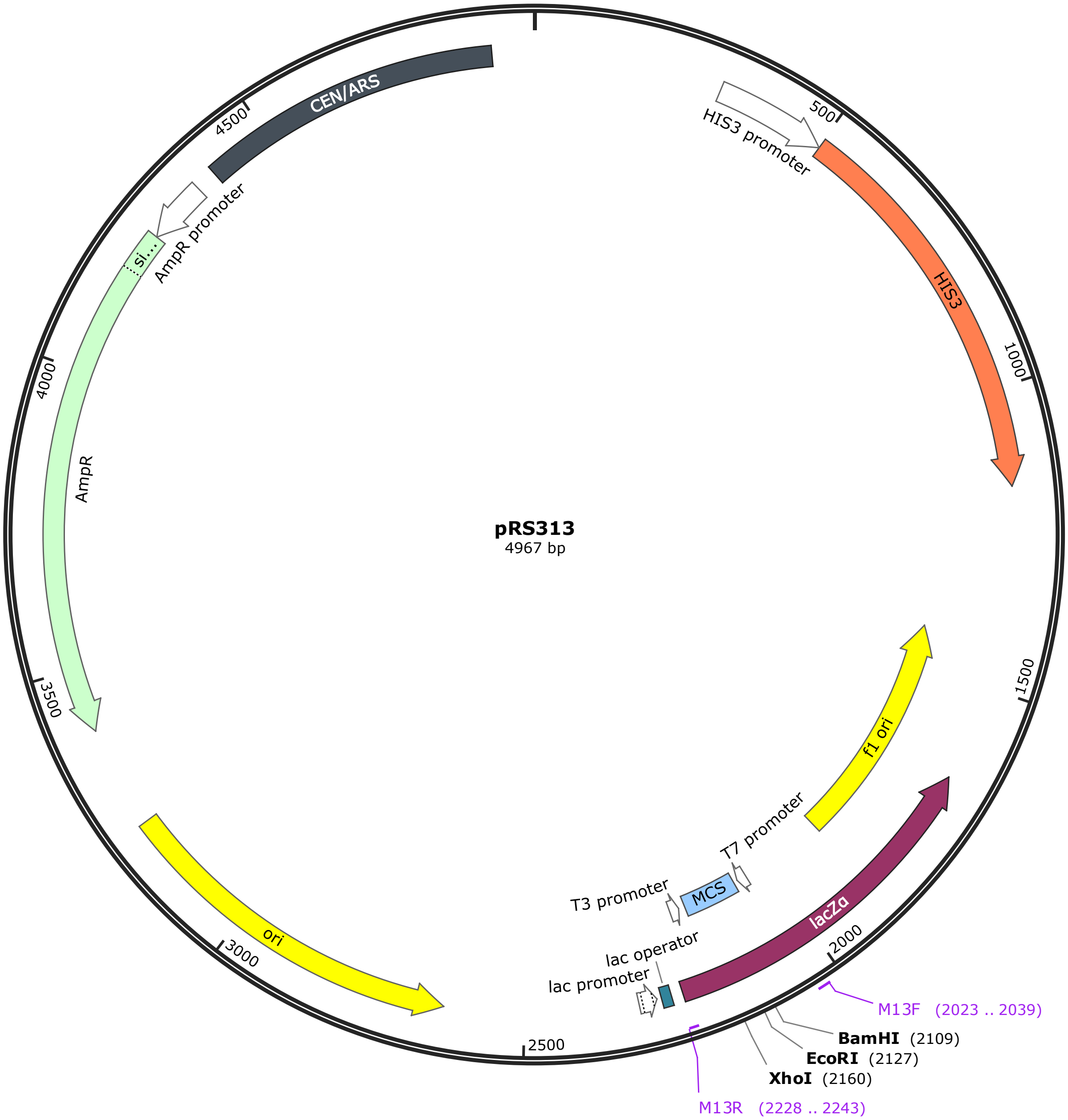

>pRS313 (4967 bp)

```
tcgcgcgtttcggtgatgacggtgaaaacctctgacacatgcagctcccggagacggtcacagc
ttgtctgtaagcggatgccgggagcagacaagcccgtcagggcgcgtcagcgggtgttggcggg
tgtcggggctggcttaactatgcggcatcagagcagattgtactgagagtgcaccataattccg
ttttaagagcttggtgagcgctaggagtcactgccaggtatcgtttgaacacggcattagtcag
ggaagtcataacacagtcctttcccgcaattttctttttctattactcttggcctcctctagta
cactctatatttttttatgcctcggtaatgattttcattttttttttttccacctagcggatgac
tcttttttttttcttagcgattggcattatcacataatgaattatacattatataaagtaatgtg
atttcttcgaagaatatactaaaaaatgagcaggcaagataaacgaaggcaaagatgacagagc
agaaagccctagtaaagcgtattacaaatgaaaccaagattcagattgcgatctctttaaaggg
```

tggtcccctagcgatagagcactcgatcttcccagaaaaagaggcagaagcagtagcagaacag
gccacacaatcgcaagtgattaacgtccacacaggtatagggtttctggaccatatgatacatg
ctctggccaagcattccggctggtcgctaatcgttgagtgcattggtgacttacacatagacga
ccatcacaccactgaagactgcgggattgctctcggtcaagcttttaaagaggccctactggcg
cgtggagtaaaaaggtttggatcaggatttgcgcctttggatgaggcactttccagagcggtgg
tagatctttcgaacaggccgtacgcagttgtcgaacttggtttgcaaagggagaaagtaggaga
tctctcttgcgagatgatcccgcattttcttgaaagctttgcagaggctagcagaattaccctc
cacgttgattgtctgcgaggcaagaatgatcatcaccgtagtgagagtgcgttcaaggctcttg
cggttgccataagagaagccacctcgcccaatggtaccaacgatgttccctccaccaaaggtgt
tcttatgtagtgacaccgattatttaaagctgcagcatacgatatatatacatgtgtatatatg
tatacctatgaatgtcagtaagtatgtatacgaacagtatgatactgaagatgacaaggtaatg
catcattctatacgtgtcattctgaacgaggcgcgctttcctttttcttttgctttttcttt
ttttttctcttgaactcgacggatcatatgcggtgtgaaataccgcacagatgcgtaaggagaa
aataccgcatcaggaaattgtaaacgttaatattttgttaaaattcgcgttaaatttttgttaa
atcagctcattttttaaccaataggccgaaatcggcaaaatcccttataaatcaaaagaataga
ccgagatagggttgagtgttgttccagtttggaacaagagtccactattaaagaacgtggactc
caacgtcaaagggcgaaaaaccgtctatcagggcgatggcccactacgtgaaccatcaccctaa
tcaagttttttggggtcgaggtgccgtaaagcactaaatcggaaccctaaagggagcccccgat
ttagagcttgacggggaaagccggcgaacgtggcgagaaaggaagggaagaaagcgaaaggagc
gggcgctagggcgctggcaagtgtagcggtcacgctgcgcgtaaccaccacacccgccgcgctt
aatgcgccgctacagggcgcgtcgcgccattcgccattcaggctgcgcaactgttgggaagggc
gatcggtgcgggcctcttcgctattacgccagctggcgaagggggatgtgctgcaaggcgatt
aagttgggtaacgccagggttttcccagtcacgacgttgtaaaacgacggccagtgaattgtaa
tacgactcactatagggcgaattggagctccaccgcggtggcggccgctctagaactagtggat
cccccgggctgcaggaattcgatatcaagcttatcgataccgtcgacctcgagggggggcccgg
tacccagcttttgttccctttagtgagggttaattccgagcttggcgtaatcatggtcatagct
gtttcctgtgtgaaattgttatccgctcacaattccacacaacataggagccggaagcataaag
tgtaaagcctggggtgcctaatgagtgaggtaactcacattaattgcgttgcgctcactgcccg
ctttccagtcgggaaacctgtcgtgccagctgcattaatgaatcggccaacgcgcggggagagg
cggtttgcgtattgggcgctcttccgcttcctcgctcactgactcgctgcgctcggtcgttcgg
ctgcggcgagcggtatcagctcactcaaaggcggtaatacggttatccacagaatcaggggata
acgcaggaaagaacatgtgagcaaaaggccagcaaaaggccaggaaccgtaaaaaggccgcgtt
gctggcgtttttccataggctcggcccccctgacgagcatcacaaaaatcgacgctcaagtcag
aggtggcgaaacccgacaggactataaagataccaggcgtttccccctggaagctccctcgtgc
gctctcctgttccgaccctgccgcttaccggatacctgtccgcctttctcccttcgggaagcgt
ggcgctttctcaatgctcacgctgtaggtatctcagttcggtgtaggtcgttcgctccaagctg
ggctgtgtgcacgaaccccccgttcagcccgaccgctgcgccttatccggtaactatcgtcttg
agtccaacccggtaagacacgacttatcgccactggcagcagccactggtaacaggattagcag
agcgaggtatgtaggcggtgctacagagttcttgaagtggtggcctaactacggctacactaga
aggacagtatttggtatctgcgctctgctgaagccagttaccttcggaaaaagagttggtagct
cttgatccggcaaacaaaccaccgctggtagcggtggtttttttgtttgcaagcagcagattac
gcgcagaaaaaaaggatctcaagaagatcctttgatcttttctacggggtctgacgctcagtgg
aacgaaaactcacgttaagggattttggtcatgagattatcaaaaaggatcttcacctagatcc
ttttaaattaaaaatgaagttttaaatcaatctaaagtatatatgagtaaacttggtctgacag
ttaccaatgcttaatcagtgaggcacctatctcagcgatctgtctatttcgttcatccatagtt
gcctgactgccccgtcgtgtagataactacgatacgggagggcttaccatctggccccagtgctg
caatgataccgcgagacccacgctcaccggctccagatttatcagcaataaaccagccagccgg

aagggccgagcgcagaagtggtcctgcaactttatccgcctccatccagtctattaattgttgc
cgggaagctagagtaagtagttcgccagttaatagtttgcgcaacgttgttgccattgctacag
gcatcgtggtgtcacgctcgtcgtttggtatggcttcattcagctccggttcccaacgatcaag
gcgagttacatgatcccccatgttgtgaaaaaaagcggttagctccttcggtcctccgatcgtt
gtcagaagtaagttggccgcagtgttatcactcatggttatggcagcactgcataattctctta
ctgtcatgccatccgtaagatgcttttctgtgactggtgagtactcaaccaagtcattctgaga
atagtgtatgcggcgaccgagttgctcttgcccggcgtcaatacgggataataccgcgccacat
agcagaactttaaaagtgctcatcattggaaaacgttcttcggggcgaaaactctcaaggatct
taccgctgttgagatccagttcgatgtaacccactcgtgcacccaactgatcttcagcatcttt
tactttcaccagcgtttctgggtgagcaaaaacaggaaggcaaaatgccgcaaaaaagggaata
agggcgacacggaaatgttgaatactcatactcttcctttttcaatattattgaagcatttatc
agggttattgtctcatgagcggatacatatttgaatgtatttagaaaaataaacaaataggggt
tccgcgcacatttccccgaaaagtgccacctgggtccttttcatcacgtgctataaaaataatt
ataatttaaatttttaatataaatatataaattaaaaatagaaagtaaaaaaagaaattaaag
aaaaaatagtttttgttttccgaagatgtaaaagactctagggggatcgccaacaaatactacc
ttttatcttgctcttcctgctctcaggtattaatgccgaattgtttcatcttgtctgtgtagaa
gaccacacacgaaaatcctgtgattttacattttacttatcgttaatcgaatgtatatctattt
aatctgcttttcttgtctaataaatatatatgtaaagtacgctttttgttgaaattttttaaac
ctttgtttatttttttttcttcattccgtaactcttctaccttctttatttactttctaaaatc
caaatacaaaacataaaaataaataaacacagagtaaattcccaaattattccatcattaaaag
atacgaggcgcgtgtaagttacaggcaagcgatccgtcctaagaaaccattattatcatgacat
taacctataaaaataggcgtatcacgaggccctttcgtc

Figure S3 – pRHis Plasmid Map and Sequence

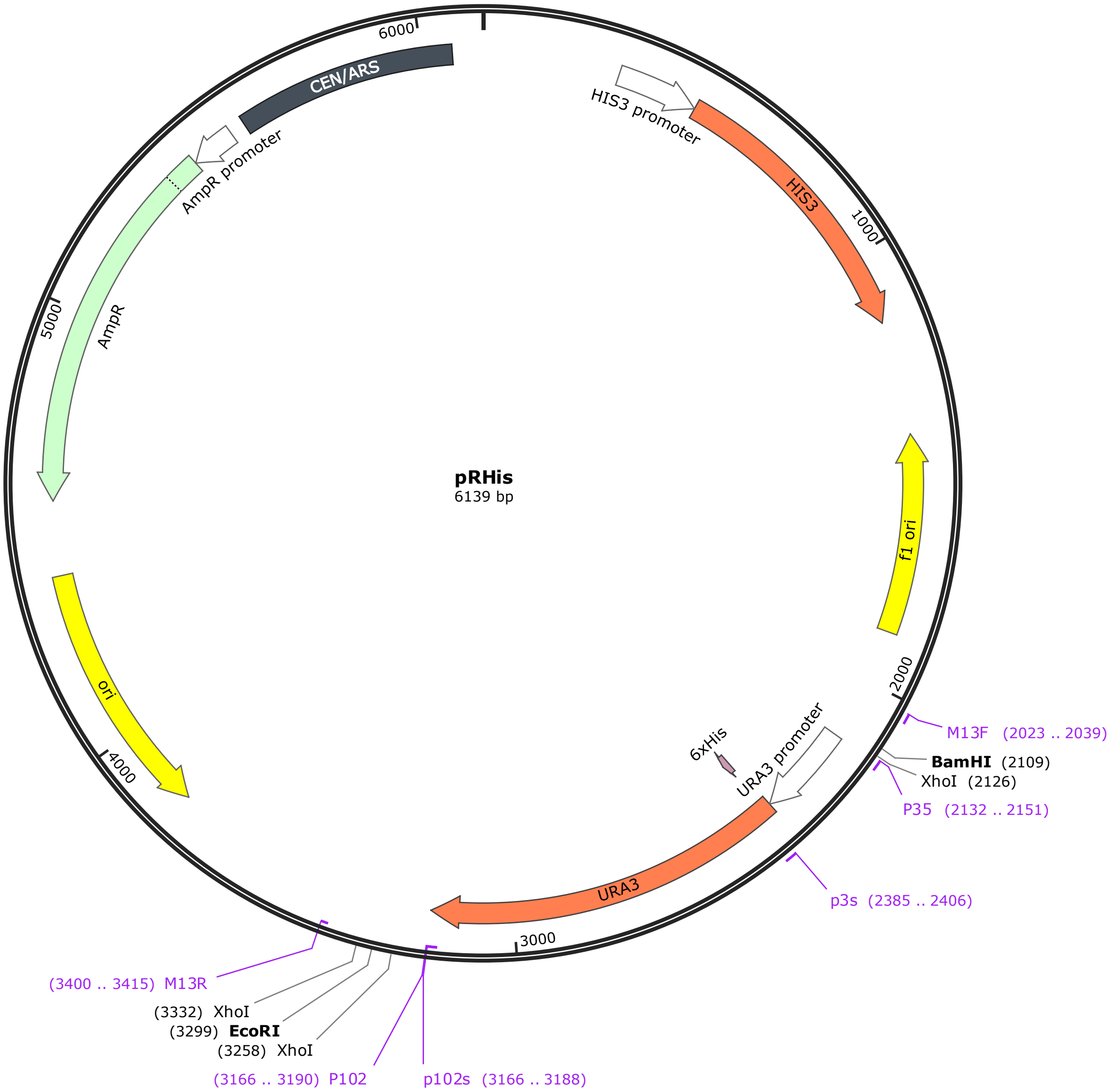


```
>pRHis  (6139 bp)
tcgcgcgtttcggtgatgacggtgaaaacctctgacacatgcagctcccggagacggtcacagc
ttgtctgtaagcggatgccgggagcagacaagcccgtcagggcgcgtcagcgggtgttggcggg
tgtcggggctggcttaactatgcggcatcagagcagattgtactgagagtgcaccataattccg
ttttaagagcttggtgagcgctaggagtcactgccaggtatcgtttgaacacggcattagtcag
ggaagtcataacacagtcctttcccgcaattttctttttctattactcttggcctcctctagta
cactctatatttttttatgcctcggtaatgattttcatttttttttttccacctagcggatgac
tctttttttttcttagcgattggcattatcacataatgaattatacattatataaagtaatgtg
atttcttcgaagaatatactaaaaaatgagcaggcaagataaacgaaggcaaagatgacagagc
```

agaaagccctagtaaagcgtattacaaatgaaaccaagattcagattgcgatctctttaaaggg
tggtcccctagcgatagagcactcgatcttcccagaaaaagaggcagaagcagtagcagaacag
gccacacaatcgcaagtgattaacgtccacacaggtatagggtttctggaccatatgatacatg
ctctggccaagcattccggctggtcgctaatcgttgagtgcattggtgacttacacatagacga
ccatcacaccactgaagactgcgggattgctctcggtcaagcttttaaagaggccctactggcg
cgtggagtaaaaaggtttggatcaggatttgcgcctttggatgaggcactttccagagcggtgg
tagatctttcgaacaggccgtacgcagttgtcgaacttggtttgcaaagggagaaagtaggaga
tctctcttgcgagatgatcccgcattttcttgaaagctttgcagaggctagcagaattaccctc
cacgttgattgtctgcgaggcaagaatgatcatcaccgtagtgagagtgcgttcaaggctcttg
cggttgccataagagaagccacctcgcccaatggtaccaacgatgttccctccaccaaaggtgt
tcttatgtagtgacaccgattatttaaagctgcagcatacgatatatatacatgtgtatatatg
tatacctatgaatgtcagtaagtatgtatacgaacagtatgatactgaagatgacaaggtaatg
catcattctatacgtgtcattctgaacgaggcgcgctttcctttttttctttttgctttttcttt
ttttttctcttgaactcgacggatcatatgcggtgtgaaataccgcacagatgcgtaaggagaa
aataccgcatcaggaaattgtaaacgttaatattttgttaaaattcgcgttaaatttttgttaa
atcagctcattttttaaccaataggccgaaatcggcaaaatcccttataaatcaaaagaataga
ccgagatagggttgagtgttgttccagtttggaacaagagtccactattaaagaacgtggactc
caacgtcaaagggcgaaaaaccgtctatcagggcgatggcccactacgtgaaccatcaccctaa
tcaagttttttggggtcgaggtgccgtaaagcactaaatcggaaccctaaagggagcccccgat
ttagagcttgacggggaaagccggcgaacgtggcgagaaaggaagggaagaaagcgaaaggagc
gggcgctagggcgctggcaagtgtagcggtcacgctgcgcgtaaccaccacacccgccgcgctt
aatgcgccgctacagggcgcgtcgcgccattcgccattcaggctgcgcaactgttgggaagggc
gatcggtgcgggcctcttcgctattacgccagctggcgaaggggggatgtgctgcaaggcgatt
aagttgggtaacgccagggttttcccagtcacgacgttgtaaaacgacggccagtgaattgtaa
tacgactcactatagggcgaattggagctccaccgcggtggcggccgctctagaactagtgGAT
CCGATGACCCAATCTCGAGGCTTTTCAATTCAATTCATCTTTTTTTTTTTTTGTTCTTTTTTTTTG
ATTCCGGTTTCTTTGAAATTTTTTTGATTCGGTAATCTCCGAGCAGAAGGAAGAACGAAGGAAG
GAGCACAGACTTAGATTGGTATATATACGCATATGTGGTGTTGAAGAAACATGAAATTGCCCAG
TATTCTTAACCCAACTGCACAGAACAAAAACCTGCAGGAAACGAAGATAAATCATGGGATCACA
CCACCACCACCACCACATGTCGAAAGCTACATATAAGGAACGTGCTGCTACTCATCCTAGTCCT
GTTGCTGCCAAGCTATTTAATATCATGCACGAAAAGCAAACAAACTTGTGTGCTTCATTGGATG
TTCGTACCACCAAGGAATTACTGGAGTTAGTTGAAGCATTAGGTCCCAAAATTTGTTTACTAAA
AACACATGTGGATATCTTGACTGATTTTTCCATGGAGGGCACAGTTAAGCCGCTAAAGGCATTA
TCCGCCAAGTACAATTTTTTACTCTTCGAAGACAGAAAATTTGCTGACATTGGTAATACAGTCA
AATTGCAGTACTCTGCGGGTGTATACAGAATAGCAGAATGGGCAGACATTACGAATGCACACGG
TGTGGTGGGCCCAGGTATTGTTAGCGGTTTGAAGCAGGCGGCAGAAGAAGTAACAAAGGAACCT
AGAGGCCTTTTGATGTTAGCAGAATTGTCATGCAAGGGCTCCCTATCTACTGGAGAATATACTA
AGGGTACTGTTGACATTGCGAAGAGCGACAAAGATTTTGTTATCGGCTTTATTGCTCAAAGAGA
CATGGGTGGAAGAGATGAAGGTTACGATTGGTTGATTATGACACCCGGTGTGGGTTTAGATGAC
AAGGGAGACGCATTGGGTCAACAGTATAGAACCGTGGATGATGTGGTCTCTACAGGATCTGACA
TTATTATTGTTGGAAGAGGACTATTTGCAAAGGGAAGGGATGCTAAGGTAGAGGGTGAACGTTA
CAGAAAAGCAGGCTGGGAAGCATATTTGAGAAGATGCGGCCAGCAAAACTAAAAAACTGTATTA
TAAGTAAATGCATGTATACTAAACTCACAAATTAGAGCTTCAATTTAATTATATCAGCTCGAGA
TTGGATCTAGATGCATTCGCGAGGTACCGAGCTCGaattcgatatcaagcttatcgataccgtc
gacctcgagggggggcccggtacccagcttttgttccctttagtgagggttaattccgagcttg
gcgtaatcatggtcatagctgtttcctgtgtgaaattgttatccgctcacaattccacacaaca
taggagccggaagcataaagtgtaaagcctggggtgcctaatgagtgaggtaactcacattaat

tgcgttgcgctcactgcccgctttccagtcgggaaacctgtcgtgccagctgcattaatgaatc
ggccaacgcgcggggagaggcggtttgcgtattgggcgctcttccgcttcctcgctcactgact
cgctgcgctcggtcgttcggctgcggcgagcggtatcagctcactcaaaggcggtaatacggtt
atccacagaatcaggggataacgcaggaaagaacatgtgagcaaaaggccagcaaaaggccagg
aaccgtaaaaaggccgcgttgctggcgtttttccataggctcggcccccctgacgagcatcaca
aaaatcgacgctcaagtcagaggtggcgaaacccgacaggactataaagataccaggcgtttcc
ccctggaagctccctcgtgcgctctcctgttccgaccctgccgcttaccggatacctgtccgcc
tttctcccttcgggaagcgtggcgctttctcaatgctcacgctgtaggtatctcagttcggtgt
aggtcgttcgctccaagctgggctgtgtgcacgaaccccccgttcagcccgaccgctgcgcctt
atccggtaactatcgtcttgagtccaacccggtaagacacgacttatcgccactggcagcagcc
actggtaacaggattagcagagcgaggtatgtaggcggtgctacagagttcttgaagtggtggc
ctaactacggctacactagaaggacagtatttggtatctgcgctctgctgaagccagttacctt
cggaaaaagagttggtagctcttgatccggcaaacaaaccaccgctggtagcggtggtttttt
gtttgcaagcagcagattacgcgcagaaaaaaaggatctcaagaagatcctttgatcttttcta
cggggtctgacgctcagtggaacgaaaactcacgttaagggattttggtcatgagattatcaaa
aaggatcttcacctagatccttttaaattaaaaatgaagttttaaatcaatctaaagtatatat
gagtaaacttggtctgacagttaccaatgcttaatcagtgaggcacctatctcagcgatctgtc
tatttcgttcatccatagttgcctgactgcccgtcgtgtagataactacgatacgggagggctt
accatctggccccagtgctgcaatgataccgcgagacccacgctcaccggctccagatttatca
gcaataaaccagccagccggaagggccgagcgcagaagtggtcctgcaactttatccgcctcca
tccagtctattaattgttgccgggaagctagagtaagtagttcgccagttaatagtttgcgcaa
cgttgttgccattgctacaggcatcgtggtgtcacgctcgtcgtttggtatggcttcattcagc
tccggttcccaacgatcaaggcgagttacatgatcccccatgttgtgaaaaaaagcggttagct
ccttcggtcctccgatcgttgtcagaagtaagttggccgcagtgttatcactcatggttatggc
agcactgcataattctcttactgtcatgccatccgtaagatgcttttctgtgactggtgagtac
tcaaccaagtcattctgagaatagtgtatgcggcgaccgagttgctcttgcccggcgtcaatac
gggataataccgcgccacatagcagaactttaaaagtgctcatcattggaaaacgttcttcggg
gcgaaaactctcaaggatcttaccgctgttgagatccagttcgatgtaacccactcgtgcaccc
aactgatcttcagcatcttttactttcaccagcgtttctgggtgagcaaaaacaggaaggcaaa
atgccgcaaaaaagggaataagggcgacacggaaatgttgaatactcatactcttcctttttca
atattattgaagcatttatcagggttattgtctcatgagcggatacatatttgaatgtatttag
aaaaataaacaaataggggttccgcgcacatttccccgaaaagtgccacctgggtccttttcat
cacgtgctataaaaataattataatttaaattttttaatataaatatataaattaaaaatagaa
agtaaaaaaagaaattaaagaaaaaatagtttttgttttccgaagatgtaaaagactctagggg
gatcgccaacaaatactaccttttatcttgctcttcctgctctcaggtattaatgccgaattgt
ttcatcttgtctgtgtagaagaccacacacgaaaatcctgtgattttacattttacttatcgtt
aatcgaatgtatatctatttaatctgcttttcttgtctaataaatatatatgtaaagtacgctt
tttgttgaaattttttaaacctttgtttatttttttttcttcattccgtaactcttctaccttc
tttatttactttctaaaatccaaatacaaaacataaaaataaataaacacagagtaaattccca
aattattccatcattaaaagatacgaggcgcgtgtaagttacaggcaagcgatccgtcctaaga
aaccattattatcatgacattaacctataaaaataggcgtatcacgaggccctttcgtc

Figure S4 – pRExt Plasmid Map and Sequence

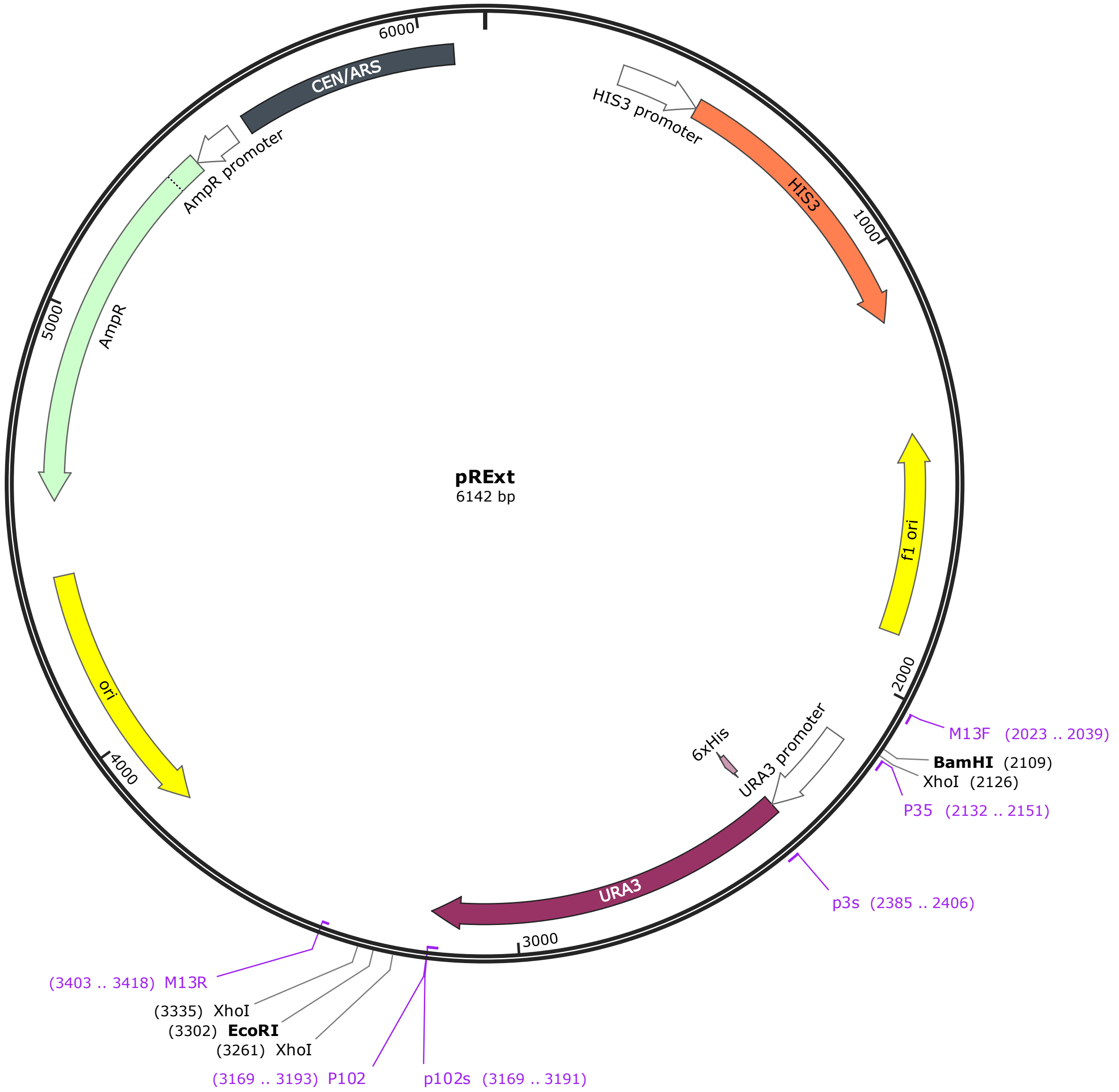


```
>pRExt  (6142 bp)
tcgcgcgtttcggtgatgacggtgaaaacctctgacacatgcagctcccggagacggtcacagc
ttgtctgtaagcggatgccgggagcagacaagcccgtcagggcgcgtcagcgggtgttggcggg
tgtcggggctggcttaactatgcggcatcagagcagattgtactgagagtgcaccataattccg
ttttaagagcttggtgagcgctaggagtcactgccaggtatcgtttgaacacggcattagtcag
ggaagtcataacacagtcctttcccgcaattttctttttctattactcttggcctcctctagta
cactctatatttttttatgcctcggtaatgattttcatttttttttttccacctagcggatgac
tctttttttttcttagcgattggcattatcacataatgaattatacattatataaagtaatgtg
atttcttcgaagaatatactaaaaaatgagcaggcaagataaacgaaggcaaagatgacagagc
```

agaaagccctagtaaagcgtattacaaatgaaaccaagattcagattgcgatctctttaaaggg
tggtcccctagcgatagagcactcgatcttcccagaaaaagaggcagaagcagtagcagaacag
gccacacaatcgcaagtgattaacgtccacacaggtatagggtttctggaccatatgatacatg
ctctggccaagcattccggctggtcgctaatcgttgagtgcattggtgacttacacatagacga
ccatcacaccactgaagactgcgggattgctctcggtcaagcttttaaagaggccctactggcg
cgtggagtaaaaaggtttggatcaggatttgcgcctttggatgaggcactttccagagcggtgg
tagatctttcgaacaggccgtacgcagttgtcgaacttggtttgcaaagggagaaagtaggaga
tctctcttgcgagatgatcccgcattttcttgaaagctttgcagaggctagcagaattaccctc
cacgttgattgtctgcgaggcaagaatgatcatcaccgtagtgagagtgcgttcaaggctcttg
cggttgccataagagaagccacctcgcccaatggtaccaacgatgttccctccaccaaaggtgt
tcttatgtagtgacaccgattatttaaagctgcagcatacgatatatatacatgtgtatatatg
tatacctatgaatgtcagtaagtatgtatacgaacagtatgatactgaagatgacaaggtaatg
catcattctatacgtgtcattctgaacgaggcgcgctttcctttttcttttgctttttcttt
ttttttctcttgaactcgacggatcatatgcggtgtgaaataccgcacagatgcgtaaggagaa
aataccgcatcaggaaattgtaaacgttaatattttgttaaaattcgcgttaaatttttgttaa
atcagctcattttttaaccaataggccgaaatcggcaaaatcccttataaatcaaaagaataga
ccgagatagggttgagtgttgttccagtttggaacaagagtccactattaaagaacgtggactc
caacgtcaaagggcgaaaaaccgtctatcagggcgatggcccactacgtgaaccatcaccctaa
tcaagttttttggggtcgaggtgccgtaaagcactaaatcggaaccctaaagggagcccccgat
ttagagcttgacggggaaagccggcgaacgtggcgagaaaggaagggaagaaagcgaaaggagc
gggcgctagggcgctggcaagtgtagcggtcacgctgcgcgtaaccaccacacccgccgcgctt
aatgcgccgctacagggcgcgtcgcgccattcgccattcaggctgcgcaactgttgggaagggc
gatcggtgcgggcctcttcgctattacgccagctggcgaaggggggatgtgctgcaaggcgatt
aagttgggtaacgccagggttttcccagtcacgacgttgtaaaacgacggccagtgaattgtaa
tacgactcactatagggcgaattggagctccaccgcggtggcggccgctctagaactagtgGAT
CCGATGACCCAATCTCGAGGCTTTTCAATTCAATTCATCTTTTTTTTTTTTTGTTCTTTTTTTTTG
ATTCCGGTTTCTTTGAAATTTTTTTGATTCGGTAATCTCCGAGCAGAAGGAAGAACGAAGGAAG
GAGCACAGACTTAGATTGGTATATATACGCATATGTGGTGTTGAAGAAACATGAAATTGCCCAG
TATTCTTAACCCAACTGCACAGAACAAAAACCTGCAGGAAACGAAGATAAATCATGGGATCACA
CCACCACCACCACCACATGTCGAAAGCTACATATAAGGAACGTGCTGCTACTCATCCTAGTCCT
GTTGCTGCCAAGCTATTTAATATCATGCACGAAAAGCAAACAAACTTGTGTGCTTCATTGGATG
TTCGTACCACCAAGGAATTACTGGAGTTAGTTGAAGCATTAGGTCCCAAAATTTGTTTACTAAA
AACACATGTGGATATCTTGACTGATTTTTCCATGGAGGGCACAGTTAAGCCGCTAAAGGCATTA
TCCGCCAAGTACAATTTTTTACTCTTCGAAGACAGAAAATTTGCTGACATTGGTAATACAGTCA
AATTGCAGTACTCTGCGGGTGTATACAGAATAGCAGAATGGGCAGACATTACGAATGCACACGG
TGTGGTGGGCCCAGGTATTGTTAGCGGTTTGAAGCAGGCGGCAGAAGAAGTAACAAAGGAACCT
AGAGGCCTTTTGATGTTAGCAGAATTGTCATGCAAGGCCTCCGGCCTATCTACTGGAGAATATA
CTAAGGGTACTGTTGACATTGCGAAGAGCGACAAAGATTTTGTTATCGGCTTTATTGCTCAAAG
AGACATGGGTGGAAGAGATGAAGGTTACGATTGGTTGATTATGACACCCGGTGTGGGTTTAGAT
GACAAGGGAGACGCATTGGGTCAACAGTATAGAACCGTGGATGATGTGGTCTCTACAGGATCTG
ACATTATTATTGTTGGAAGAGGACTATTTGCAAAGGGAAGGGATGCTAAGGTAGAGGGTGAACG
TTACAGAAAAGCAGGCTGGGAAGCATATTTGAGAAGATGCGGCCAGCAAAACTAAAAAACTGTA
TTATAAGTAAATGCATGTATACTAAACTCACAAATTAGAGCTTCAATTTAATTATATCAGCTCG
AGATTGGATCTAGATGCATTCGCGAGGTACCGAGCTCGaattcgatatcaagcttatcgatacc
gtcgacctcgagggggggcccggtacccagcttttgttccctttagtgagggttaattccgagc
ttggcgtaatcatggtcatagctgtttcctgtgtgaaattgttatccgctcacaattccacaca
acataggagccggaagcataaagtgtaaagcctggggtgcctaatgagtgaggtaactcacatt

aattgcgttgcgctcactgcccgctttccagtcgggaaacctgtcgtgccagctgcattaatga
atcggccaacgcgcggggagaggcggtttgcgtattgggcgctcttccgcttcctcgctcactg
actcgctgcgctcggtcgttcggctgcggcgagcggtatcagctcactcaaaggcggtaatacg
gttatccacagaatcaggggataacgcaggaaagaacatgtgagcaaaaggccagcaaaaggcc
aggaaccgtaaaaaggccgcgttgctggcgtttttccataggctcggcccccctgacgagcatc
acaaaaatcgacgctcaagtcagaggtggcgaaacccgacaggactataaagataccaggcgtt
tccccctggaagctccctcgtgcgctctcctgttccgaccctgccgcttaccggatacctgtcc
gcctttctcccttcgggaagcgtggcgctttctcaatgctcacgctgtaggtatctcagttcgg
tgtaggtcgttcgctccaagctgggctgtgtgcacgaaccccccgttcagcccgaccgctgcgc
cttatccggtaactatcgtcttgagtccaacccggtaagacacgacttatcgccactggcagca
gccactggtaacaggattagcagagcgaggtatgtaggcggtgctacagagttcttgaagtggt
ggcctaactacggctacactagaaggacagtatttggtatctgcgctctgctgaagccagttac
cttcggaaaaagagttggtagctcttgatccggcaaacaaaccaccgctggtagcggtggtttt
tttgtttgcaagcagcagattacgcgcagaaaaaaaggatctcaagaagatcctttgatctttt
ctacggggtctgacgctcagtggaacgaaaactcacgttaagggattttggtcatgagattatc
aaaaaggatcttcacctagatccttttaaattaaaaatgaagttttaaatcaatctaaagtata
tatgagtaaacttggtctgacagttaccaatgcttaatcagtgaggcacctatctcagcgatct
gtctatttcgttcatccatagttgcctgactgcccgtcgtgtagataactacgatacgggaggg
cttaccatctggccccagtgctgcaatgataccgcgagacccacgctcaccggctccagattta
tcagcaataaaccagccagccggaagggccgagcgcagaagtggtcctgcaactttatccgcct
ccatccagtctattaattgttgccgggaagctagagtaagtagttcgccagttaatagtttgcg
caacgttgttgccattgctacaggcatcgtggtgtcacgctcgtcgtttggtatggcttcattc
agctccggttcccaacgatcaaggcgagttacatgatcccccatgttgtgaaaaaaagcggtta
gctccttcggtcctccgatcgttgtcagaagtaagttggccgcagtgttatcactcatggttat
ggcagcactgcataattctcttactgtcatgccatccgtaagatgcttttctgtgactggtgag
tactcaaccaagtcattctgagaatagtgtatgcggcgaccgagttgctcttgcccggcgtcaa
tacgggataataccgcgccacatagcagaactttaaaagtgctcatcattggaaaacgttcttc
ggggcgaaaactctcaaggatcttaccgctgttgagatccagttcgatgtaacccactcgtgca
cccaactgatcttcagcatcttttactttcaccagcgtttctgggtgagcaaaaacaggaaggc
aaaatgccgcaaaaaagggaataagggcgacacggaaatgttgaatactcatactcttcctttt
tcaatattattgaagcatttatcagggttattgtctcatgagcggatacatatttgaatgtatt
tagaaaaataaacaaataggggttccgcgcacatttccccgaaaagtgccacctgggtcctttt
catcacgtgctataaaaataattataatttaaattttttaatataaatatataaattaaaaata
gaaagtaaaaaaagaaattaaagaaaaaatagtttttgttttccgaagatgtaaaagactctag
ggggatcgccaacaaatactacctttttatcttgctcttcctgctctcaggtattaatgccgaat
tgtttcatcttgtctgtgtagaagaccacacacgaaaatcctgtgattttacattttacttatc
gttaatcgaatgtatatctatttaatctgcttttcttgtctaataaatatatatgtaaagtacg
ctttttgttgaaattttttaaacctttgtttatttttttttcttcattccgtaactcttctacc
ttctttatttactttctaaaatccaaatacaaaacataaaaataaataaacacagagtaaattc
ccaaattattccatcattaaaagatacgaggcgcgtgtaagttacaggcaagcgatccgtccta
agaaaccattattatcatgacattaacctataaaaataggcgtatcacgaggccctttcgtc

Figure S5 – pRInt Plasmid Map and Sequence

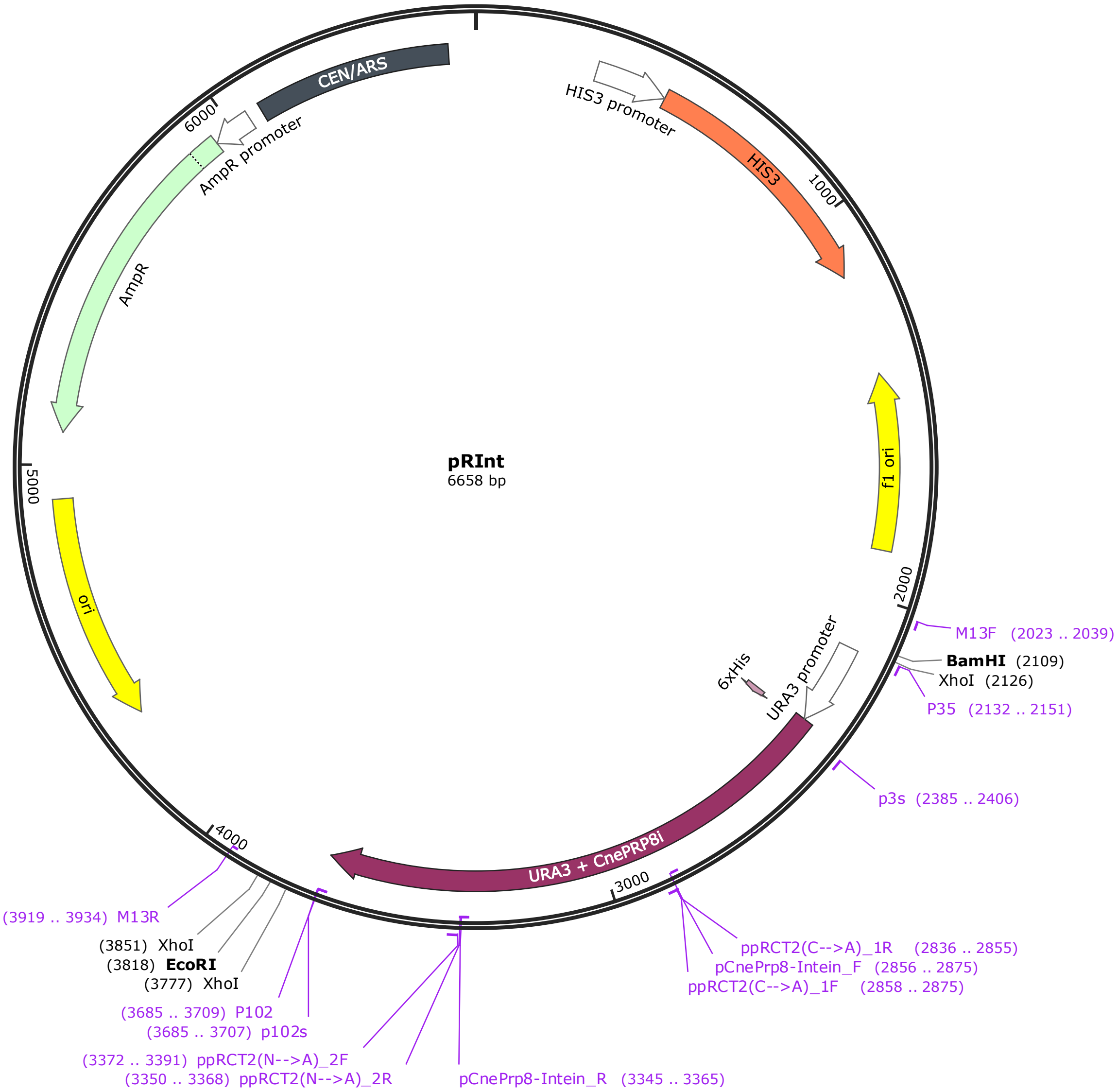


```
>pRInt  (6658 bp)
tcgcgcgtttcggtgatgacggtgaaaacctctgacacatgcagctcccggagacggtcacagc
ttgtctgtaagcggatgccgggagcagacaagcccgtcagggcgcgtcagcgggtgttggcggg
tgtcggggctggcttaactatgcggcatcagagcagattgtactgagagtgcaccataattccg
ttttaagagcttggtgagcgctaggagtcactgccaggtatcgtttgaacacggcattagtcag
ggaagtcataacacagtcctttcccgcaattttctttttctattactcttggcctcctctagta
cactctatatttttttatgcctcggtaatgattttcatttttttttttccacctagcggatgac
tctttttttttcttagcgattggcattatcacataatgaattatacattatataaagtaatgtg
atttcttcgaagaatatactaaaaaatgagcaggcaagataaacgaaggcaaagatgacagagc
agaaagccctagtaaagcgtattacaaatgaaaccaagattcagattgcgatctctttaaaggg
tggtcccctagcgatagagcactcgatcttcccagaaaaagaggcagaagcagtagcagaacag
gccacacaatcgcaagtgattaacgtccacacaggtatagggtttctggaccatatgatacatg
```

ctctggccaagcattccggctggtcgctaatcgttgagtgcattggtgacttacacatagacga
ccatcacaccactgaagactgcgggattgctctcggtcaagcttttaaagaggccctactggcg
cgtggagtaaaaaggtttggatcaggatttgcgcctttggatgaggcactttccagagcggtgg
tagatctttcgaacaggccgtacgcagttgtcgaacttggtttgcaaagggagaaagtaggaga
tctctcttgcgagatgatcccgcattttcttgaaagctttgcagaggctagcagaattaccctc
cacgttgattgtctgcgaggcaagaatgatcatcaccgtagtgagagtgcgttcaaggctcttg
cggttgccataagagaagccacctcgcccaatggtaccaacgatgttccctccaccaaaggtgt
tcttatgtagtgacaccgattatttaaagctgcagcatacgatatatatacatgtgtatatatg
tatacctatgaatgtcagtaagtatgtatacgaacagtatgatactgaagatgacaaggtaatg
catcattctatacgtgtcattctgaacgaggcgcgctttccttttttctttttgctttttcttt
ttttttctcttgaactcgacggatcatatgcggtgtgaaataccgcacagatgcgtaaggagaa
aataccgcatcaggaaattgtaaacgttaatattttgttaaaattcgcgttaaatttttgttaa
atcagctcattttttaaccaataggccgaaatcggcaaaatcccttataaatcaaaagaataga
ccgagatagggttgagtgttgttccagtttggaacaagagtccactattaaagaacgtggactc
caacgtcaaagggcgaaaaaccgtctatcagggcgatggcccactacgtgaaccatcaccctaa
tcaagttttttggggtcgaggtgccgtaaagcactaaatcggaaccctaaagggagcccccgat
ttagagcttgacggggaaagccggcgaacgtggcgagaaaggaagggaagaaagcgaaaggagc
gggcgctagggcgctggcaagtgtagcggtcacgctgcgcgtaaccaccacacccgccgcgctt
aatgcgccgctacagggcgcgtcgcgccattcgccattcaggctgcgcaactgttgggaagggc
gatcggtgcgggcctcttcgctattacgccagctggcgaaggggggatgtgctgcaaggcgatt
aagttgggtaacgccagggttttcccagtcacgacgttgtaaaacgacggccagtgaattgtaa
tacgactcactatagggcgaattggagctccaccgcggtggcggccgctctagaactagtgGAT
CCGATGACCCAATCTCGAGGCTTTTCAATTCAATTCATCTTTTTTTTTTTTGTTCTTTTTTTTG
ATTCCGGTTTCTTTGAAATTTTTTTGATTCGGTAATCTCCGAGCAGAAGGAAGAACGAAGGAAG
GAGCACAGACTTAGATTGGTATATATACGCATATGTGGTGTTGAAGAAACATGAAATTGCCCAG
TATTCTTAACCCAACTGCACAGAACAAAAACCTGCAGGAAACGAAGATAAATCATGGGATCACA
CCACCACCACCACCACATGTCGAAAGCTACATATAAGGAACGTGCTGCTACTCATCCTAGTCCT
GTTGCTGCCAAGCTATTTAATATCATGCACGAAAAGCAAACAAACTTGTGTGCTTCATTGGATG
TTCGTACCACCAAGGAATTACTGGAGTTAGTTGAAGCATTAGGTCCCAAAATTTGTTTACTAAA
AACACATGTGGATATCTTGACTGATTTTTCCATGGAGGGCACAGTTAAGCCGCTAAAGGCATTA
TCCGCCAAGTACAATTTTTTACTCTTCGAAGACAGAAAATTTGCTGACATTGGTAATACAGTCA
AATTGCAGTACTCTGCGGGTGTATACAGAATAGCAGAATGGGCAGACATTACGAATGCACACGG
TGTGGTGGGCCCAGGTATTGTTAGCGGTTTGAAGCAGGCGGCAGAAGAAGTAACAAAGGAACCT
AGAGGCCTTTTGATGTTAGCAGAATTGTCATGCAAGGCCTGTCTGCAGAATGGTACTCGTCTTC
TCCGTGCCGATGGCTCTGAGGTCCTTGTGGAAGATGTTCAGGAGGGCGATCAACTTCTTGGTCC
CGATGGAACGAGCAGGACGGCGAGCAAGATTGTTCGCGGCGAAGAGCGTCTCTACCGTATCAAA
ACCCATGAGGGGCTCGAAGATCTTGTCTGTACCCATAACCACATCCTTTCTATGTATAAAGAAA
GGTCTGGTTCGGAGCGAGCTCATTCTCCTAGTGCCGACCTCAGCCTCACAGACAGCCATGAGAG
AGTCGATGTGACTGTCGATGACTTTGTCCGCCTTCCTCAACAAGAGCAACAGAAGTATCAGCTT
TTCCGTTCAACTGCTTCTGTGCGACACGAGCGACCATTCACTTCTAAATTAGACACCACCTTGT
TACGCATCAATTCTATCGAGCTTGAGGACGAGCCTACGAAGTGGTCCGGTTTTGTGGTTGACAA
AGACAGTCTTTATCTTCGTCATGACTATTTGGTATTGCACAACTCCGGCCTATCTACTGGAGAA
TATACTAAGGGTACTGTTGACATTGCGAAGAGCGACAAAGATTTTGTTATCGGCTTTATTGCTC
AAAGAGACATGGGTGGAAGAGATGAAGGTTACGATTGGTTGATTATGACACCCGGTGTGGGTTT
AGATGACAAGGGAGACGCATTGGGTCAACAGTATAGAACCGTGGATGATGTGGTCTCTACAGGA
TCTGACATTATTATTGTTGGAAGAGGACTATTTGCAAAGGGAAGGGATGCTAAGGTAGAGGGTG
AACGTTACAGAAAAGCAGGCTGGGAAGCATATTTGAGAAGATGCGGCCAGCAAAACTAAAAAAC

TGTATTATAAGTAAATGCATGTATACTAAACTCACAAATTAGAGCTTCAATTTAATTATATCAG
CTCGAGATTGGATCTAGATGCATTCGCGAGGTACCGAGCTCGaattcgatatcaagcttatcga
taccgtcgacctcgagggggggcccggtacccagcttttgttccctttagtgagggttaattcc
gagcttggcgtaatcatggtcatagctgtttcctgtgtgaaattgttatccgctcacaattcca
cacaacataggagccggaagcataaagtgtaaagcctggggtgcctaatgagtgaggtaactca
cattaattgcgttgcgctcactgcccgctttccagtcgggaaacctgtcgtgccagctgcatta
atgaatcggccaacgcgcggggagaggcggtttgcgtattgggcgctcttccgcttcctcgctc
actgactcgctgcgctcggtcgttcggctgcggcgagcggtatcagctcactcaaaggcggtaa
tacggttatccacagaatcaggggataacgcaggaaagaacatgtgagcaaaaggccagcaaaa
ggccaggaaccgtaaaaaggccgcgttgctggcgtttttccataggctcggcccccctgacgag
catcacaaaaatcgacgctcaagtcagaggtggcgaaacccgacaggactataaagataccagg
cgtttccccctggaagctccctcgtgcgctctcctgttccgaccctgccgcttaccggatacct
gtccgcctttctcccttcgggaagcgtggcgctttctcaatgctcacgctgtaggtatctcagt
tcggtgtaggtcgttcgctccaagctgggctgtgtgcacgaaccccccgttcagcccgaccgct
gcgccttatccggtaactatcgtcttgagtccaacccggtaagacacgacttatcgccactggc
agcagccactggtaacaggattagcagagcgaggtatgtaggcggtgctacagagttcttgaag
tggtggcctaactacggctacactagaaggacagtatttggtatctgcgctctgctgaagccag
ttaccttcggaaaaagagttggtagctcttgatccggcaaacaaaccaccgctggtagcggtgg
tttttttgtttgcaagcagcagattacgcgcagaaaaaaaggatctcaagaagatcctttgatc
ttttctacggggtctgacgctcagtggaacgaaaactcacgttaagggattttggtcatgagat
tatcaaaaaggatcttcacctagatccttttaaattaaaaatgaagttttaaatcaatctaaag
tatatatgagtaaacttggtctgacagttaccaatgcttaatcagtgaggcacctatctcagcg
atctgtctatttcgttcatccatagttgcctgactgcccgtcgtgtagataactacgatacggg
agggcttaccatctggccccagtgctgcaatgataccgcgagacccacgctcaccggctccaga
tttatcagcaataaaccagccagccggaagggccgagcgcagaagtggtcctgcaactttatcc
gcctccatccagtctattaattgttgccgggaagctagagtaagtagttcgccagttaatagtt
tgcgcaacgttgttgccattgctacaggcatcgtggtgtcacgctcgtcgtttggtatggcttc
attcagctccggttcccaacgatcaaggcgagttacatgatcccccatgttgtgaaaaaaagcg
gttagctccttcggtcctccgatcgttgtcagaagtaagttggccgcagtgttatcactcatgg
ttatggcagcactgcataattctcttactgtcatgccatccgtaagatgcttttctgtgactgg
tgagtactcaaccaagtcattctgagaatagtgtatgcggcgaccgagttgctcttgcccggcg
tcaatacgggataataccgcgccacatagcagaactttaaaagtgctcatcattggaaaacgtt
cttcggggcgaaaactctcaaggatcttaccgctgttgagatccagttcgatgtaacccactcg
tgcacccaactgatcttcagcatcttttactttcaccagcgtttctgggtgagcaaaaacagga
aggcaaaatgccgcaaaaaagggaataagggcgacacggaaatgttgaatactcatactcttcc
tttttcaatattattgaagcatttatcagggttattgtctcatgagcggatacatatttgaatg
tatttagaaaaataaacaaataggggttccgcgcacatttccccgaaaagtgccacctgggtcc
ttttcatcacgtgctataaaaataattataatttaaattttttaatataaatatataaattaaa
aatagaaagtaaaaaaagaaattaaagaaaaaatagtttttgttttccgaagatgtaaaagact
ctagggggatcgccaacaaatactaccttttatcttgctcttcctgctctcaggtattaatgcc
gaattgtttcatcttgtctgtgtagaagaccacacacgaaaatcctgtgattttacattttact
tatcgttaatcgaatgtatatctatttaatctgcttttcttgtctaataaatatatatgtaaag
tacgcttttttgttgaaattttttaaacctttgtttattttttttttcttcattccgtaactcttc
taccttctttatttactttctaaaatccaaatacaaaacataaaaataaataaacacagagtaa
attcccaaattattccatcattaaaagatacgaggcgcgtgtaagttacaggcaagcgatccgt
cctaagaaaccattattatcatgacattaacctataaaaataggcgtatcacgaggccctttcg
tc

Figure S6 – pRMut Plasmid Map and Sequence

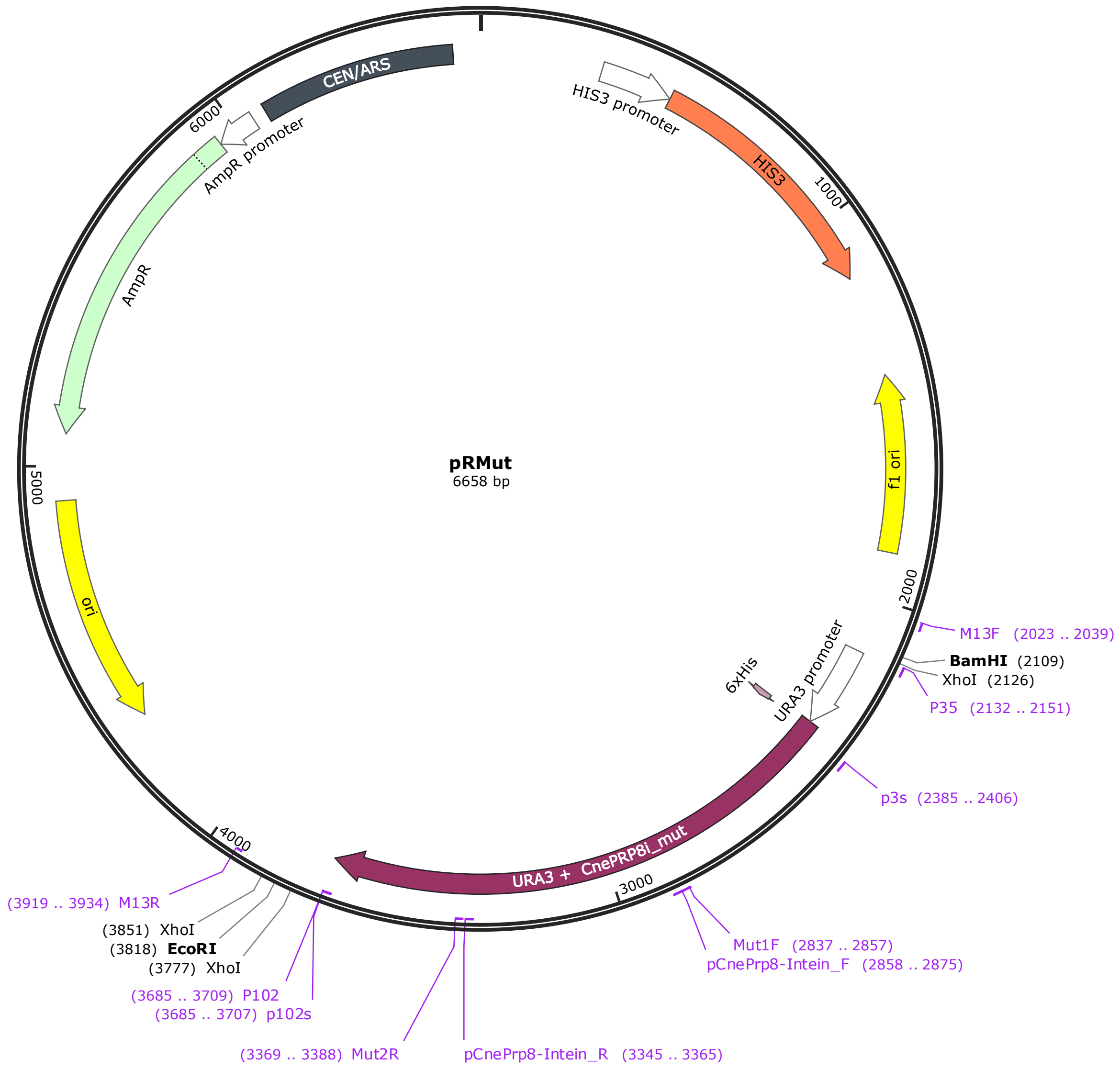


```
>pRMut  (6658 bp)
tcgcgcgtttcggtgatgacggtgaaaacctctgacacatgcagctcccggagacggtcacagc
ttgtctgtaagcggatgccgggagcagacaagcccgtcagggcgcgtcagcgggtgttggcggg
tgtcggggctggcttaactatgcggcatcagagcagattgtactgagagtgcaccataattccg
ttttaagagcttggtgagcgctaggagtcactgccaggtatcgtttgaacacggcattagtcag
ggaagtcataacacagtcctttcccgcaattttctttttctattactcttggcctcctctagta
cactctatatttttttatgcctcggtaatgattttcattttttttttttccacctagcggatgac
tctttttttttcttagcgattggcattatcacataatgaattatacattatataagtaatgtg
atttcttcgaagaatatactaaaaaatgagcaggcaagataaacgaaggcaaagatgacagagc
agaaagccctagtaaagcgtattacaaatgaaaccaagattcagattgcgatctctttaaaggg
tggtcccctagcgatagagcactcgatcttcccagaaaaagaggcagaagcagtagcagaacag
gccacacaatcgcaagtgattaacgtccacacaggtatagggtttctggaccatatgatacatg
```

ctctggccaagcattccggctggtcgctaatcgttgagtgcattggtgacttacacatagacga
ccatcacaccactgaagactgcgggattgctctcggtcaagcttttaaagaggccctactggcg
cgtggagtaaaaaggtttggatcaggatttgcgcctttggatgaggcactttccagagcggtgg
tagatctttcgaacaggccgtacgcagttgtcgaacttggtttgcaaagggagaaagtaggaga
tctctcttgcgagatgatcccgcattttcttgaaagctttgcagaggctagcagaattaccctc
cacgttgattgtctgcgaggcaagaatgatcatcaccgtagtgagagtgcgttcaaggctcttg
cggttgccataagagaagccacctcgcccaatggtaccaacgatgttccctccaccaaaggtgt
tcttatgtagtgacaccgattatttaaagctgcagcatacgatatatatacatgtgtatatatg
tatacctatgaatgtcagtaagtatgtatacgaacagtatgatactgaagatgacaaggtaatg
catcattctatacgtgtcattctgaacgaggcgcgctttcctttttttcttttttgctttttcttt
tttttctcttgaactcgacggatcatatgcggtgtgaaataccgcacagatgcgtaaggagaa
aataccgcatcaggaaattgtaaacgttaatattttgttaaaattcgcgttaaatttttgttaa
atcagctcattttttaaccaataggccgaaatcggcaaaatcccttataaatcaaaagaataga
ccgagatagggttgagtgttgttccagtttggaacaagagtccactattaaagaacgtggactc
caacgtcaaagggcgaaaaaccgtctatcagggcgatggcccactacgtgaaccatcaccctaa
tcaagttttttggggtcgaggtgccgtaaagcactaaatcggaaccctaaagggagcccccgat
ttagagcttgacggggaaagccggcgaacgtggcgagaaaggaagggaagaaagcgaaaggagc
gggcgctagggcgctggcaagtgtagcggtcacgctgcgcgtaaccaccacacccgccgcgctt
aatgcgccgctacagggcgcgtcgcgccattcgccattcaggctgcgcaactgttgggaagggc
gatcggtgcgggcctcttcgctattacgccagctggcgaaggggggatgtgctgcaaggcgatt
aagttgggtaacgccagggttttcccagtcacgacgttgtaaaacgacggccagtgaattgtaa
tacgactcactatagggcgaattggagctccaccgcggtggcggccgctctagaactagtgGAT
CCGATGACCCAATCTCGAGGCTTTTCAATTCAATTCATCTTTTTTTTTTTTTGTTCTTTTTTTTG
ATTCCGGTTTCTTTGAAATTTTTTTGATTCGGTAATCTCCGAGCAGAAGGAAGAACGAAGGAAG
GAGCACAGACTTAGATTGGTATATATACGCATATGTGGTGTTGAAGAAACATGAAATTGCCCAG
TATTCTTAACCCAACTGCACAGAACAAAAACCTGCAGGAAACGAAGATAAATCATGGATCACA
CCACCACCACCACCACATGTCGAAAGCTACATATAAGGAACGTGCTGCTACTCATCCTAGTCCT
GTTGCTGCCAAGCTATTTAATATCATGCACGAAAAGCAAACAAACTTGTGTGCTTCATTGGATG
TTCGTACCACCAAGGAATTACTGGAGTTAGTTGAAGCATTAGGTCCCAAAATTTGTTTACTAAA
AACACATGTGGATATCTTGACTGATTTTTCCATGGAGGGCACAGTTAAGCCGCTAAAGGCATTA
TCCGCCAAGTACAATTTTTTACTCTTCGAAGACAGAAAATTTGCTGACATTGGTAATACAGTCA
AATTGCAGTACTCTGCGGGTGTATACAGAATAGCAGAATGGGCAGACATTACGAATGCACACGG
TGTGGTGGGCCCAGGTATTGTTAGCGGTTTGAAGCAGGCGGCAGAAGAAGTAACAAAGGAACCT
AGAGGCCTTTTGATGTTAGCAGAATTGTCATGCAAGGCCGCTCTGCAGAATGGTACTCGTCTTC
TCCGTGCCGATGGCTCTGAGGTCCTTGTGGAAGATGTTCAGGAGGGCGATCAACTTCTTGGTCC
CGATGGAACGAGCAGGACGGCGAGCAAGATTGTTCGCGGCGAAGAGCGTCTCTACCGTATCAAA
ACCCATGAGGGGCTCGAAGATCTTGTCTGTACCCATAACCACATCCTTTCTATGTATAAAGAAA
GGTCTGGTTCGGAGCGAGCTCATTCTCCTAGTGCCGACCTCAGCCTCACAGACAGCCATGAGAG
AGTCGATGTGACTGTCGATGACTTTGTCCGCCTTCCTCAACAAGAGCAACAGAAGTATCAGCTT
TTCCGTTCAACTGCTTCTGTGCGACACGAGCGACCATTCACTTCTAAATTAGACACCACCTTGT
TACGCATCAATTCTATCGAGCTTGAGGACGAGCCTACGAAGTGGTCCGGTTTTGTGGTTGACAA
AGACAGTCTTTATCTTCGTCATGACTATTTGGTATTGCACGCTTCCGGCCTATCTACTGGAGAA
TATACTAAGGGTACTGTTGACATTGCGAAGAGCGACAAAGATTTTGTTATCGGCTTTATTGCTC
AAAGAGACATGGGTGGAAGAGATGAAGGTTACGATTGGTTGATTATGACACCCGGTGTGGGTTT
AGATGACAAGGGAGACGCATTGGGTCAACAGTATAGAACCGTGGATGATGTGGTCTCTACAGGA
TCTGACATTATTATTGTTGGAAGAGGACTATTTGCAAAGGGAAGGGATGCTAAGGTAGAGGGTG
AACGTTACAGAAAAGCAGGCTGGGAAGCATATTTGAGAAGATGCGGCCAGCAAAACTAAAAAAC

TGTATTATAAGTAAATGCATGTATACTAAACTCACAAATTAGAGCTTCAATTTAATTATATCAG
CTCGAGATTGGATCTAGATGCATTCGCGAGGTACCGAGCTCGaattcgatatcaagcttatcga
taccgtcgacctcgagggggggcccggtacccagcttttgttccctttagtgagggttaattcc
gagcttggcgtaatcatggtcatagctgtttcctgtgtgaaattgttatccgctcacaattcca
cacaacataggagccggaagcataaagtgtaaagcctggggtgcctaatgagtgaggtaactca
cattaattgcgttgcgctcactgcccgctttccagtcgggaaacctgtcgtgccagctgcatta
atgaatcggccaacgcgcggggagaggcggtttgcgtattgggcgctcttccgcttcctcgctc
actgactcgctgcgctcggtcgttcggctgcggcgagcggtatcagctcactcaaaggcggtaa
tacggttatccacagaatcaggggataacgcaggaaagaacatgtgagcaaaaggccagcaaaa
ggccaggaaccgtaaaaaggccgcgttgctggcgtttttccataggctcggcccccctgacgag
catcacaaaaatcgacgctcaagtcagaggtggcgaaacccgacaggactataaagataccagg
cgtttccccctggaagctccctcgtgcgctctcctgttccgaccctgccgcttaccggatacct
gtccgcctttctcccttcgggaagcgtggcgctttctcaatgctcacgctgtaggtatctcagt
tcggtgtaggtcgttcgctccaagctgggctgtgtgcacgaaccccccgttcagcccgaccgct
gcgccttatccggtaactatcgtcttgagtccaacccggtaagacacgacttatcgccactggc
agcagccactggtaacaggattagcagagcgaggtatgtaggcggtgctacagagttcttgaag
tggtggcctaactacggctacactagaaggacagtatttggtatctgcgctctgctgaagccag
ttaccttcggaaaaagagttggtagctcttgatccggcaaacaaaccaccgctggtagcggtgg
tttttttgtttgcaagcagcagattacgcgcagaaaaaaaggatctcaagaagatcctttgatc
ttttctacggggtctgacgctcagtggaacgaaaactcacgttaagggattttggtcatgagat
tatcaaaaaggatcttcacctagatccttttaaattaaaaatgaagttttaaatcaatctaaag
tatatatgagtaaacttggtctgacagttaccaatgcttaatcagtgaggcacctatctcagcg
atctgtctatttcgttcatccatagttgcctgactgcccgtcgtgtagataactacgatacggg
agggcttaccatctggccccagtgctgcaatgataccgcgagacccacgctcaccggctccaga
tttatcagcaataaaccagccagccggaagggccgagcgcagaagtggtcctgcaactttatcc
gcctccatccagtctattaattgttgccgggaagctagagtaagtagttcgccagttaatagtt
tgcgcaacgttgttgccattgctacaggcatcgtggtgtcacgctcgtcgtttggtatggcttc
attcagctccggttcccaacgatcaaggcgagttacatgatcccccatgttgtgaaaaaaagcg
gttagctccttcggtcctccgatcgttgtcagaagtaagttggccgcagtgttatcactcatgg
ttatggcagcactgcataattctcttactgtcatgccatccgtaagatgcttttctgtgactgg
tgagtactcaaccaagtcattctgagaatagtgtatgcggcgaccgagttgctcttgcccggcg
tcaatacgggataataccgcgccacatagcagaactttaaaagtgctcatcattggaaaacgtt
cttcggggcgaaaactctcaaggatcttaccgctgttgagatccagttcgatgtaacccactcg
tgcacccaactgatcttcagcatcttttactttcaccagcgtttctgggtgagcaaaaacagga
aggcaaaatgccgcaaaaaagggaataagggcgacacggaaatgttgaatactcatactcttcc
tttttcaatattattgaagcatttatcagggttattgtctcatgagcggatacatatttgaatg
tatttagaaaaataaacaaataggggttccgcgcacatttccccgaaaagtgccacctgggtcc
ttttcatcacgtgctataaaaataattataatttaaattttttaatataaatatataaattaaa
aatagaaagtaaaaaaagaaattaaagaaaaaatagtttttgttttccgaagatgtaaaagact
ctaggggatcgccaacaaatactaccttttatcttgctcttcctgctctcaggtattaatgcc
gaattgtttcatcttgtctgtgtagaagaccacacacgaaaatcctgtgattttacattttact
tatcgttaatcgaatgtatatctatttaatctgcttttcttgtctaataaatatatatgtaaag
tacgcttttgttgaaattttttaaacctttgtttatttttttttcttcattccgtaactcttc
taccttctttatttactttctaaaatccaaatacaaaacataaaaataaataaacacagagtaa
attcccaaattattccatcattaaaagatacgaggcgcgtgtaagttacaggcaagcgatccgt
cctaagaaaccattattatcatgacattaacctataaaaataggcgtatcacgaggccctttcg
tc

Figure S7 – Alignment of the Proteins generated by this work, highlighting its modifications

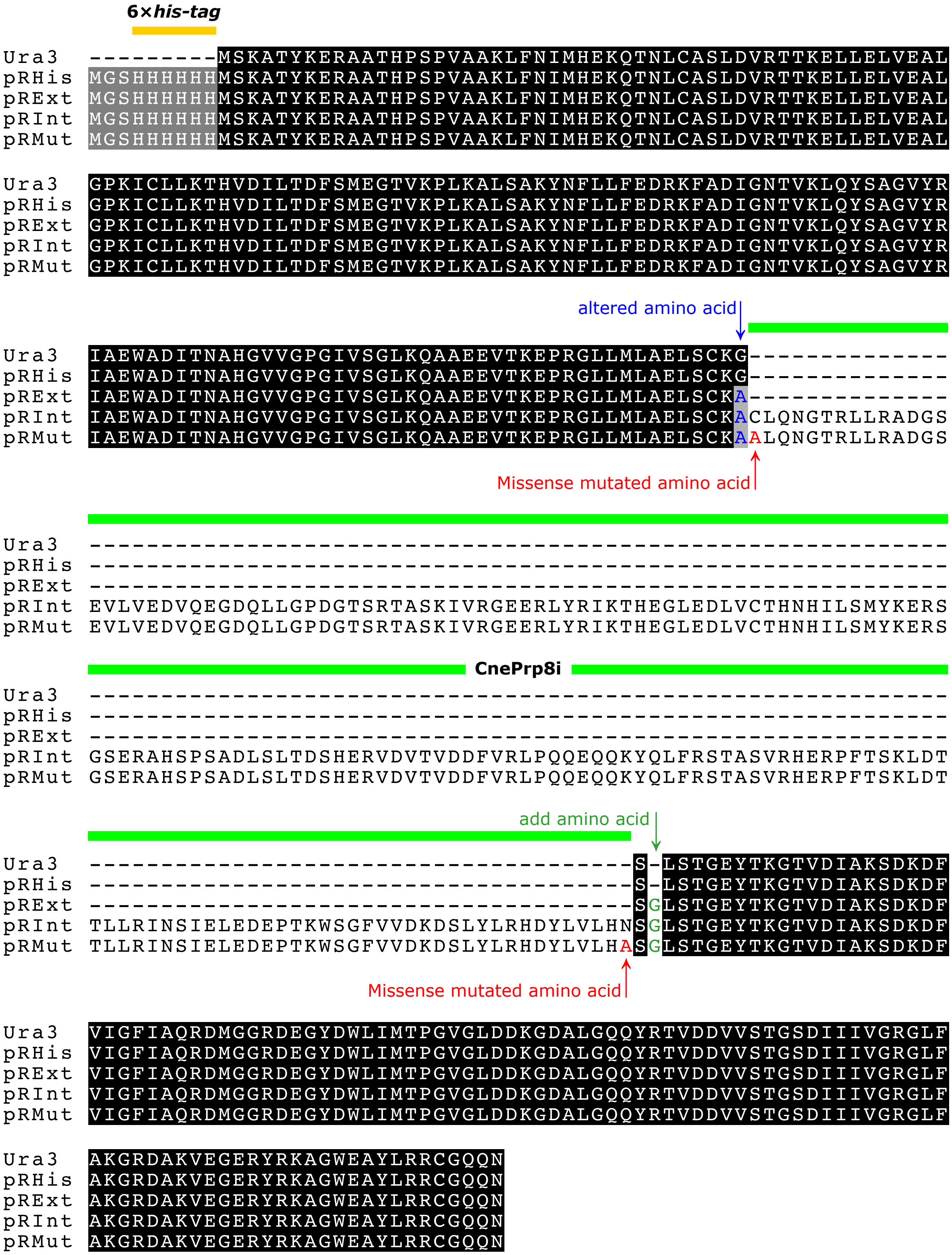

Figure S8 – Protein sequences generated by this work, highlighting its modifications

```
>Ura3
MSKATYKERAATHPSPVAAKLFNIMHEKQTNLCASLDVRTTKELLELVEALGPKICLLKTHVDI
LTDFSMEGTVKPLKALSAKYNFLLFEDRKFADIGNTVKLQYSAGVYRIAEWADITNAHGVVGPG
IVSGLKQAAEEVTKEPRGLLMLAELSCKGSLSTGEYTKGTVDIAKSDKDFVIGFIAQRDMGGRD
EGYDWLIMTPGVGLDDKGDALGQQYRTVDDVVSTGSDIIIVGRGLFAKGRDAKVEGERYRKAGW
EAYLRRCGQQN*

>pRHis
MGSHHHHHHMSKATYKERAATHPSPVAAKLFNIMHEKQTNLCASLDVRTTKELLELVEALGPKI
CLLKTHVDILTDFSMEGTVKPLKALSAKYNFLLFEDRKFADIGNTVKLQYSAGVYRIAEWADIT
NAHGVVGPGIVSGLKQAAEEVTKEPRGLLMLAELSCKGSLSTGEYTKGTVDIAKSDKDFVIGFI
AQRDMGGRDEGYDWLIMTPGVGLDDKGDALGQQYRTVDDVVSTGSDIIIVGRGLFAKGRDAKVE
GERYRKAGWEAYLRRCGQQN*

>pRExt
MGSHHHHHHMSKATYKERAATHPSPVAAKLFNIMHEKQTNLCASLDVRTTKELLELVEALGPKI
CLLKTHVDILTDFSMEGTVKPLKALSAKYNFLLFEDRKFADIGNTVKLQYSAGVYRIAEWADIT
NAHGVVGPGIVSGLKQAAEEVTKEPRGLLMLAELSCKASGLSTGEYTKGTVDIAKSDKDFVIGF
IAQRDMGGRDEGYDWLIMTPGVGLDDKGDALGQQYRTVDDVVSTGSDIIIVGRGLFAKGRDAKV
EGERYRKAGWEAYLRRCGQQN*

>pRInt
MGSHHHHHHMSKATYKERAATHPSPVAAKLFNIMHEKQTNLCASLDVRTTKELLELVEALGPKI
CLLKTHVDILTDFSMEGTVKPLKALSAKYNFLLFEDRKFADIGNTVKLQYSAGVYRIAEWADIT
NAHGVVGPGIVSGLKQAAEEVTKEPRGLLMLAELSCKACLQNGTRLLRADGSEVLVEDVQEGDQ
LLGPDGTSRTASKIVRGEERLYRIKTHEGLEDLVCTHNHILSMYKERSGSERAHSPSADLSLTD
SHERVDVTVDDFVRLPQQEQQKYQLFRSTASVRHERPFTSKLDTTLLRINSIELEDEPTKWSGF
VVDKDSLYLRHDYLVLHNSGLSTGEYTKGTVDIAKSDKDFVIGFIAQRDMGGRDEGYDWLIMTP
GVGLDDKGDALGQQYRTVDDVVSTGSDIIIVGRGLFAKGRDAKVEGERYRKAGWEAYLRRCGQQ
N*

>pRMut
MGSHHHHHHMSKATYKERAATHPSPVAAKLFNIMHEKQTNLCASLDVRTTKELLELVEALGPKI
CLLKTHVDILTDFSMEGTVKPLKALSAKYNFLLFEDRKFADIGNTVKLQYSAGVYRIAEWADIT
NAHGVVGPGIVSGLKQAAEEVTKEPRGLLMLAELSCKAALQNGTRLLRADGSEVLVEDVQEGDQ
LLGPDGTSRTASKIVRGEERLYRIKTHEGLEDLVCTHNHILSMYKERSGSERAHSPSADLSLTD
SHERVDVTVDDFVRLPQQEQQKYQLFRSTASVRHERPFTSKLDTTLLRINSIELEDEPTKWSGF
VVDKDSLYLRHDYLVLHASGLSTGEYTKGTVDIAKSDKDFVIGFIAQRDMGGRDEGYDWLIMTP
GVGLDDKGDALGQQYRTVDDVVSTGSDIIIVGRGLFAKGRDAKVEGERYRKAGWEAYLRRCGQQ
N*
```
